# Supplementary material for: Morphological Adaptation in the Jejunal Mucosa after Iso-Caloric High-Fat versus High-Carbohydrate Diets in Healthy Volunteers: Data from a Randomized Crossover Study
Source: Nutrients. 2022 Oct 4;14(19):4123. doi: 10.3390/nu14194123 (PMC9572503; doi:10.3390/nu14194123)
Supplement: Supplementary file 1 [file nutrients-14-04123-s001.zip › Table S1.pdf]

Table S1. Ultrastructural analysis of mitochondria following diet intervention.

	**High carbonate diet (HCD)**				**High fat diet (HFD)**			
	**TS**	**TI**	**BS**	**BI**	**TS**	**TI**	**BS**	**BI**
**Mean area (μm^2) of cytoplasm**	126±7	38±3	107±7	25±2	107±8	40±3	93±13	25±3
**Number of granules/mitochondria**	0.49±0.1	0.53±0.1	0.3±0.1	0.25±0.04	0.55±0.1	0.45±0.1	0.39±0.1	0.36±0.1

TS, top region supranuclear; TI, top region infranuclear; BS, base region supranuclear; BI, base region infranuclear. Mean±SEM.
