# Supplementary figures and images for: Morphological Adaptation in the Jejunal Mucosa after Iso-Caloric High-Fat versus High-Carbohydrate Diets in Healthy Volunteers: Data from a Randomized Crossover Study
Source: Nutrients. 2022 Oct 4;14(19):4123. doi: 10.3390/nu14194123 (PMC9572503; doi:10.3390/nu14194123)

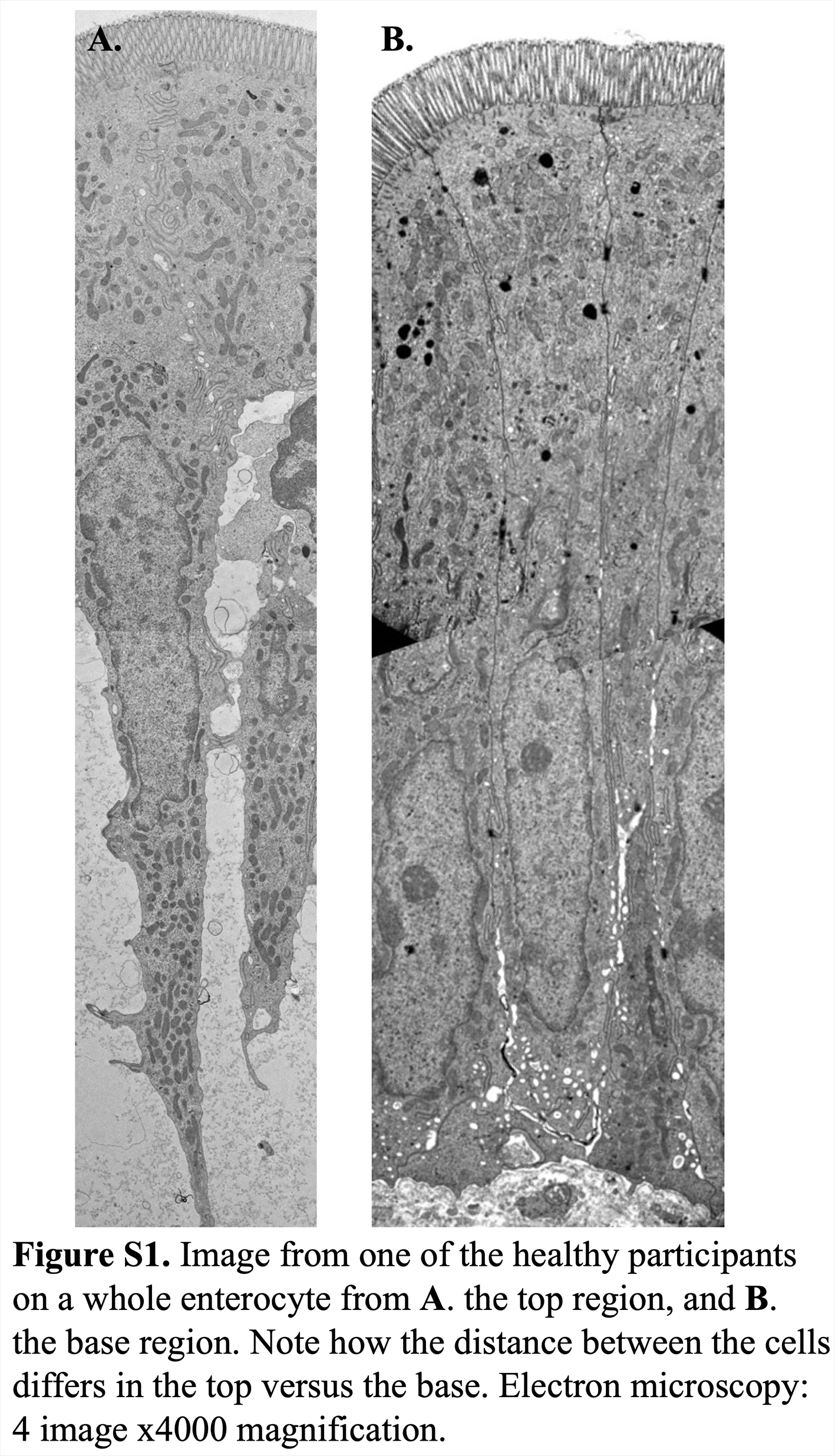

Supplement: Supplementary file 1 [file nutrients-14-04123-s001.zip › Figure S1.tiff]

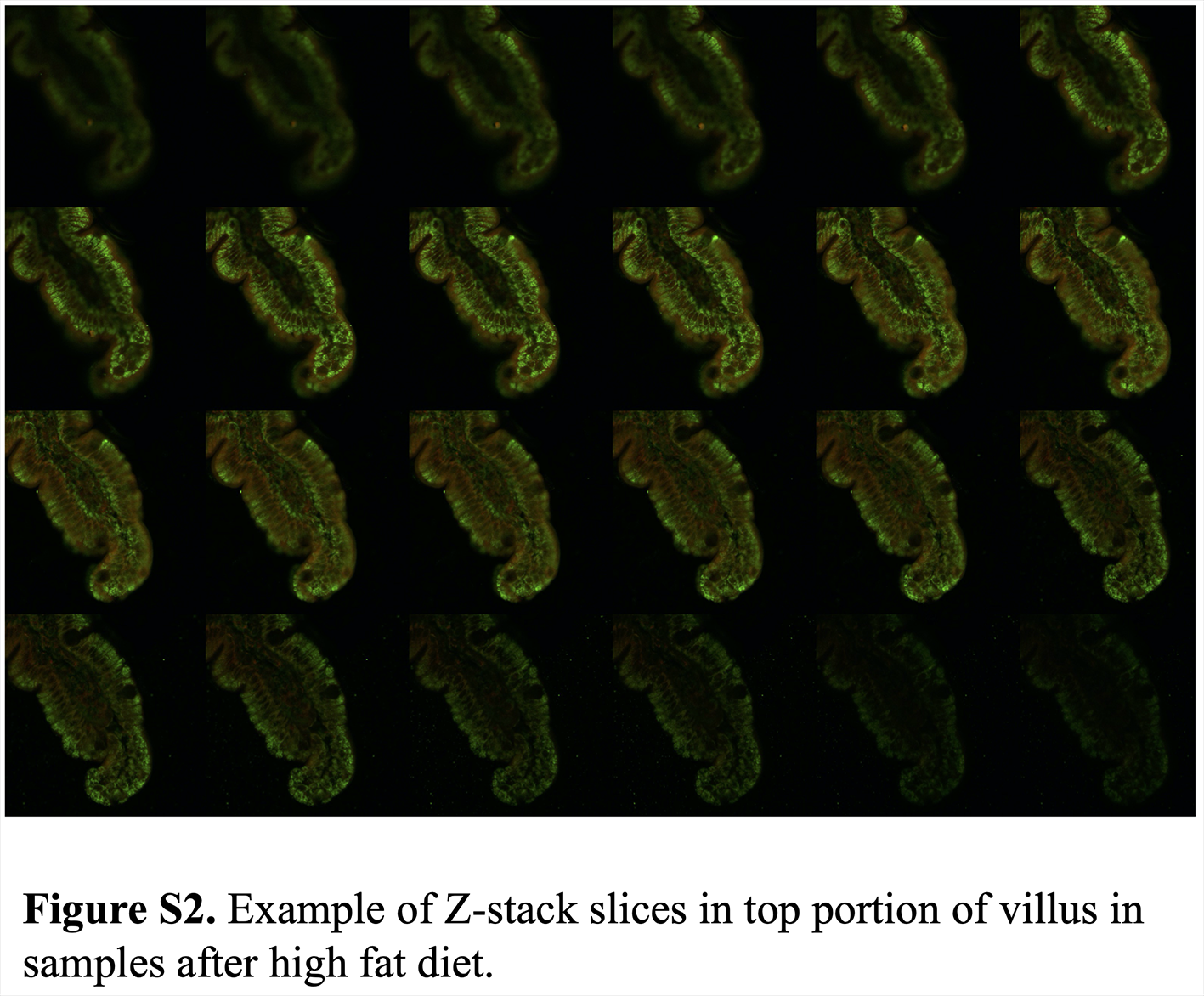

Supplement: Supplementary file 1 [file nutrients-14-04123-s001.zip › Figure S2.tiff]
